# Associations between Non-Essential Trace Elements in Women’s Biofluids and IVF Outcomes in Euploid Single-Embryo Transfer Cycles

**DOI:** 10.3390/jox14030062

**Published:** 2024-08-08

**Authors:** Roberto Gonzalez-Martin, Andrea Palomar, Silvia Perez-Deben, Stefania Salsano, Alicia Quiñonero, Laura Caracena, Isabel Rucandio, Rocio Fernandez-Saavedra, Rodolfo Fernandez-Martinez, Estefania Conde-Vilda, Alberto J. Quejido, Juan Giles, Carmen Vidal, Jose Bellver, Francisco Dominguez

**Affiliations:** 1 IVI-RMA Global Research Alliance, IVI Foundation, Instituto de Investigación Sanitaria La Fe (IIS La Fe), 46026 Valencia, Spain; roberto.gonzalez@ivirma.com (R.G.-M.); andrea.palomar@ivirma.com (A.P.); alicia.quinonero@ivirma.com (A.Q.); laura.caracena@ivirma.com (L.C.); juan.giles@ivirma.com (J.G.); carmina.vidal@ivirma.com (C.V.); jose.bellver@ivirma.com (J.B.); 2 IVI-RMA Global Research Alliance, IVI-RMA Valencia, 46015 Valencia, Spain; 3 Unit of Mass Spectrometry and Geochemical Applications, Chemistry Division, Department of Technology, Centro de Investigaciones Energéticas, Medioambientales y Tecnológicas (CIEMAT), 28040 Madrid, Spain; isabel.rucandio@ciemat.es (I.R.); rocio.fernandez@ciemat.es (R.F.-S.); rodolfo.fernandez@ciemat.es (R.F.-M.); estefania.conde@ciemat.es (E.C.-V.); alberto.quejido@ciemat.es (A.J.Q.); 4 Department of Pediatrics, Obstetrics and Gynecology, Faculty of Medicine of Valencia, 46010 Valencia, Spain

**Keywords:** non-essential trace elements, IVF outcomes, ovarian response, biofluids, ICP-MS, Tricell DMA 80

## Abstract

Previous studies have found inconsistent associations between heavy metals and metalloids (cadmium, lead, mercury, and arsenic), and reproductive outcomes. The biofluid concentrations of ten non-essential trace elements (Hg, Pb, As, Ba, Sr, Rb, Cs, Sn, Ni, and Co) were evaluated in 51 Spanish women undergoing ICSI, PGT-A, and SET/FET. Nine out of ten non-essential elements were detectable in follicular fluid, whole blood, and urine collected the day of vaginal oocyte retrieval (VOR) and the day of embryo transfer and then analyzed by ICP-MS or Tricell DMA-80 for mercury. Elevated mercury and strontium concentrations in follicular fluid were associated with poor ovarian response and preimplantation outcomes. Worst preimplantation outcomes were also identified in women with elevated whole-blood strontium or mercury, urinary arsenic, barium, and tin the day of VOR. High concentrations of urinary rubidium on VOR day were linked with enhanced fertilization and blastocyst development. Excessive titanium in whole blood was associated with lower odds of implantation, clinical pregnancy, and achieving a live birth in a given IVF cycle. Excessive urinary arsenic on the day of embryo transfer was associated with lower odds of live birth. Although these preliminary results need to be confirmed in larger populations, distinguishing organic and inorganic element forms, our findings show that some non-essential elements have a detrimental impact on human IVF outcomes.

## 1. Introduction

Anthropogenic activities have led to an excessive release of trace elements in the environment. Non-essential trace elements lack biological functions and are toxic at low concentrations [1,2,3]. Exposure to non-essential trace elements (also referred to as metals and metalloids) is widespread, with arsenic, cadmium, lead, and mercury present among the World Health Organization (WHO)’s top ten chemicals of greatest public health concern [1,2]. There is no or incomplete information available for non-essential trace elements like strontium, barium, titanium, tin, cesium, nickel, and cobalt. Chronic exposure to classically studied non-essential trace elements has been linked to the development of various pathologies, such as cancer, cardiovascular disease, renal disease, and neurocognitive defects, in addition to negative reproductive outcomes [2,4,5]. Notably, there are conflicting results among studies linking non-essential trace elements with in vitro fertilization (IVF) outcomes [6,7,8,9,10,11,12,13,14,15,16,17,18], potentially due to geographical and cultural factors or variable assisted reproductive technologies (ARTs) among clinics.

In this study, our aim was to examine the associations between the concentrations of non-essential trace elements, measured in follicular fluid (FF), whole blood (all collected on vaginal oocyte retrieval (VOR) day), and urine (collected both on the day of VOR and embryo transfer [ET]) and the IVF outcomes of a population of women undergoing an ART treatment with intracytoplasmic sperm injection (ICSI) and pre-implantation genetic screening for aneuploidy (PGT-A) prior to single, frozen, euploid embryo transfer (SET/FET).

## 2. Materials and Methods

### 2.1. Study Population

In this prospective single-center pilot study performed at IVI-RMA Valencia (Spain), we enrolled 51 women (between 18 and 42 years of age) undergoing ICSI with PGT-A and SET/FET. Recruitment took place between September 2018 and November 2019. Women were excluded if they had partners with severe male-factor infertility, uterine or endometrial abnormalities, endometrium less than 7 mm thick on the day of embryo transfer, thrombophilia, untreated endocrine or systemic disorders, or karyotype abnormalities. Eligible participants received the same standard clinical and laboratory care as they would have outside the study.

### 2.2. Collection of Follicular Fluid, Whole Blood, and Urine Samples

The collection of whole blood, urine, and follicular fluid samples was performed in a similar way to previous studies [19]. In brief, whole-blood samples were extracted by venipuncture in EDTA tubes on the day of VOR. Samples (*n* = 40) and then were aliquoted, and stored at −80 °C. Urine samples were obtained from the first morning urine of both the VOR (*n* = 50) and the ET (*n* = 27) days. These samples were centrifuged at 500× *g* for 7 min. The supernatants were aliquoted and stored at −80 °C. After oocyte retrieval, the remaining FF (*n* = 29), once the cumulus–oocyte complexes were isolated, were pooled, centrifuged at 1000× *g* for 3 min, aliquoted, and stored at −80 °C. For sample processing and preservation, trace element-free plastics were employed. Field blanks were obtained and preserved to assess possible contamination. The samples were transported on dry ice, initially to the IVI Foundation (Valencia, Spain) for processing, and subsequently to the Mass Spectrometry and Geochemical Applications Unit at CIEMAT (Madrid, Spain), for trace element quantification. Upon reaching CIEMAT, the samples were conserved at −80 °C until they could be analyzed.

### 2.3. Quantification of Non-Essential Trace Elements by ICP-MS

Non-essential trace elements concentrations (Pb, As, Ba, Sr, Rb, Cs, Sn, Ni, and Co) in FF, whole blood, and urine were measured using inductively coupled plasma–mass spectrometry (ICP-MS), as we previously reported [10,19,20]. The analysis was performed with an i-CapRQ mass spectrometer (Thermo Fisher Scientific, Madrid, Spain), which features a quadrupole analyzer and a dual-mode secondary electron multiplier (SEM) detection system. The trace elements were assessed using Collision Cell Technology (CCT) in kinetic energy discrimination mode to reduce polyatomic interferences from the biological samples.

For the quantification of mercury, a direct mercury analysis system (Tricell DMA-80 instrument, Milestone, Sorisole, Italy) was employed to analyze in triplicate the unprocessed samples, in accordance with the EPA 7473 method recommendations [21].

Urinary trace element concentrations were corrected for creatinine concentration to compensate the urinary dilution. Creatinine was measured using a commercial kit (Creatinine Parameter Assay Kit, KGE005, Bio-Techne R&D Systems, Madrid, Spain).

### 2.4. Clinical Management and Outcome Assessment

Data on the baseline characteristics of the participants were obtained from the electronic medical record. Additional demographic variables (education level, race/ethnicity, and smoking status) were extracted from a questionnaire. Serum estradiol concentrations of the ovulation trigger administration day were quantified in the clinic by automated electrochemiluminescence immunoassay.

The controlled ovarian stimulation (COS) of all participants was carried out with a gonadotropin-releasing hormone (GnRH) antagonist protocol. The clinician adjusted the hormone doses administered based on the participant’s ovarian reserve. Once the follicles had reached a size between 15 and 22 mm, final oocyte maturation was induced via the administration of human chorionic gonadotropin (hCG) and/or a GnRH agonist trigger. Thirty-six hours later, an ultrasound-guided VOR was performed [22,23,24].

The oocytes were decumulated, and the number of total and mature (metaphase II (MII)) oocytes was assessed. ICSI was applied as a fertilization method both to homogenize procedures and as part of the PGT-A protocol. Eighteen hours after insemination, the number of successfully fertilized embryos was evaluated. Embryos were cultured up to the blastocyst stage in a sequential culture medium. PGT-A was performed on a trophectoderm biopsy obtained before the embryo was vitrified [22,23,24].

The transfer of a single euploid embryo was performed after an endometrial preparation with estradiol and progesterone [22,23,24]. The thawed blastocysts were transferred into the uterine cavity with an ultrasound-guided catheter.

The evaluation of the clinical IVF outcomes was performed according to internal standardized protocols. Implantation was defined as serum hCG levels >6 mIU/mL approximately 14 days after embryo transfer. Clinical pregnancy was defined after ultrasound confirmation. Live birth was defined as delivery after 24 weeks of gestation.

### 2.5. Statistical Analysis

All statistical analyses were conducted using R software (version 3.6.2). The “tableone” package [25] was used to present the baseline demographic and reproductive characteristics of the participants. Baseline characteristics were presented as median ± interquartile ranges (IQRs) or percentages. To examine the non-essential trace element relationships between and within each biological matrix, Spearman correlation matrices were created with the “corrplot” package [26].

The evaluation of associations between non-essential trace element concentrations and IVF outcomes was performed using multivariate generalized linear models. For continuous variables (e.g., trigger day E2 concentrations), mean differences were estimated using a Gaussian distribution. In the case of discrete quantitative variables (e.g., oocyte yield, relative proportion of mature oocytes (offset by the total number of oocytes retrieved), relative proportion of fertilized oocytes (offset by the total number of inseminated oocytes), relative proportion of blastocyst (offset by the total number of fertilized embryos), and relative proportion of euploid embryos (offset by the total number of tested embryos)), mean differences or relative proportions were estimated using a Poisson distribution. Non-essential trace element concentrations were logarithmically transformed and modeled as continuous variables. The estimate of linear associations was presented as the increment between the 20th and 80th percentiles. Finally, the likelihood of having an embryo transfer, the likelihood of implantation, clinical pregnancy, or a live newborn per embryo transfer treatment and the likelihood of a live newborn per treatment initiated (termed reproductive success) were calculated as odds ratios (ORs) with a binomial distribution, using the “questionr” package [27].

We presented the adjusted population marginal means for all model covariates to facilitate interpretation of the results. Models were adjusted for age and BMI (both modeled as continuous), as potential confounders associated with both non-essential trace element exposure and reproductive outcomes [28]. A *p* < 0.05 was considered significant in all cases.

## 3. Results

### 3.1. Baseline Characteristics

Both the demographic and reproductive characteristics of the participants are listed in Table 1. All participants self-reported a similar nutritional pattern, with no nutritional supplementation.

### 3.2. Non-Essential Trace Element Distribution among Biofluids

The distributions of non-essential trace element concentrations in FF, whole blood, and urine (both VOR and ET), and the proportion of samples below the limit of detection are shown in Table 2. We excluded for further analysis non-essential trace elements detected less than 50% of the samples (Table 2).

There were a few significant correlations between the levels of non-essential trace elements in FF, blood, and urine (Supplementary Figure S1). There was a moderate positive correlation between the levels of mercury in whole blood and urine the day of VOR (r = 0.47) (Supplementary Figure S1A). A strong positive correlation was observed for arsenic between biofluids obtained during VOR (r = 0.63 for FF and blood; r = 0.74 for FF and urine; and r = 0.65 for blood and urine) (Supplementary Figure S1C). Strontium levels in FF and blood were highly correlated (r = 0.62), while blood and urine from VOR day were weakly correlated (r = 0.39) (Supplementary Figure S1E). There was a stronger correlation between cesium in both types of urine samples than in blood and urine obtained on VOR day (r = 0.58 vs. r = 0.39) (Supplementary Figure S1G).

Evaluating the correlations between non-essential trace element concentrations within biofluids identified significant moderate positive correlations between strontium and rubidium (r = 0.45) and between lead and barium (r = 0.47), along with moderate negative correlations between rubidium and barium (r = −0.46) in FF (Supplementary Figure S2A). In blood, we observed significant mild-to-moderate positive correlations between cesium, mercury, and arsenic (r = 0.51 for Hg-As, r = 0.45 for Cs-As, and r = 0.39 for Cs-Hg) and a mild correlation between cesium and strontium (r = 0.39) (Supplementary Figure S2B). In urine obtained on VOR day, there was a significantly strong positive correlation between rubidium and cesium (r = 0.62); moderate-to-strong positive correlations between lead, barium, and strontium (r = 0.69 between Ba and Sr, r = 0.58 between Ba and Pb, and r = 0.41 between Pb and Sr); and mercury and cesium levels were weakly correlated (r = 0.39). In contrast, significant moderate negative correlations were observed between rubidium with barium or strontium in VOR day urine (r = −0.4 and r = −0.43, respectively; Figure S2C). Finally, in urine obtained on ET day, we also observed strong positive correlations between rubidium and cesium (r = 0.65) and moderate correlations between barium and strontium (r = 0.58) (Supplementary Figure S2D).

### 3.3. Associations between Non-Essential Trace Element Concentrations and Ovarian Response Outcomes

The associations between the log-transformed trace element concentrations and ovarian response outcomes were analyzed using models adjusted for age and BMI. The results are presented as the relative proportions’ mean differences (95% CI) between the 20th and 80th percentiles.

**Figure 1:**
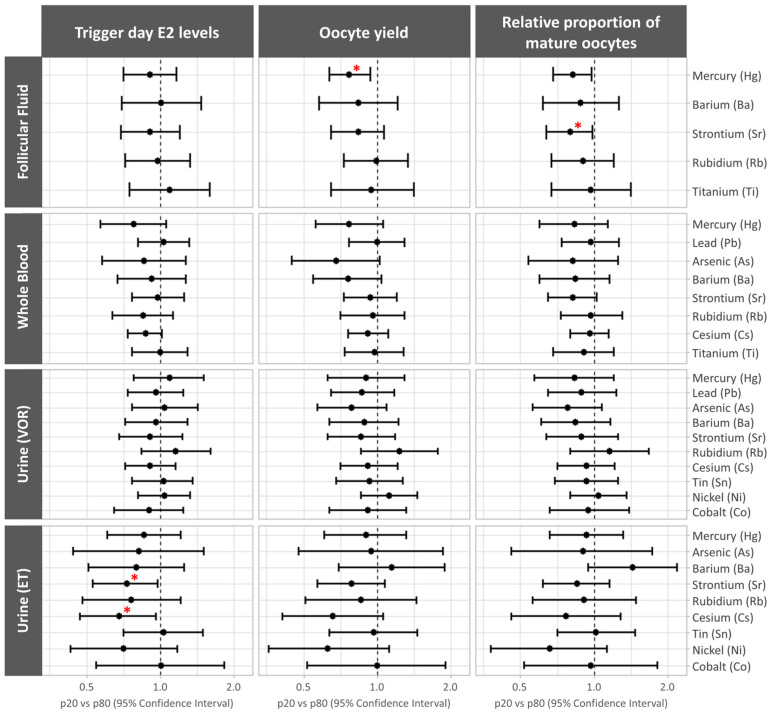
Forest plots representing the mean differences between the 20th and 80th percentiles (p20 vs. p80) (95% confidence interval) for ovarian response-related outcomes by non-essential trace element concentrations. The mean differences are presented for trigger day estradiol (E2) levels, oocyte yield, and the relative proportion of mature (MII) oocytes across the non-essential trace elements quantified in each biological matrix. Data were adjusted for age and BMI (both continuous). * *p* < 0.05. ET, embryo transfer; VOR, vaginal oocyte retrieval.

Higher FF mercury levels were associated with significantly lower oocyte yields and fewer mature oocytes (p20 vs. p80 (95% CI): 0.77 (0.64, 0.94), *p* trend = 0.01 and 0.82 (0.68, 0.98), *p* trend = 0.03, respectively). The FF strontium concentrations were negatively correlated with the relative proportion of mature oocytes (p20 vs. p80 (95% CI): 0.80 (0.64, 0.99), *p* trend = 0.04). We did not observe associations between the levels of non-essential trace elements in whole blood and urine obtained on the day of VOR and the ovarian stimulation-response outcomes. Finally, higher concentrations of strontium and cesium in ET day urine tended to be associated with lower trigger day estradiol levels (p20 vs. p80 (95% CI): 0.73 (0.53, 0.98), *p* trend = 0.04; and 0.68 (0.47, 0.96), *p* trend = 0.03, respectively) (Figure 1 and Supplementary Table S1).

In this population, no statistically significant associations were found between the remaining trace elements and the biological matrices.

### 3.4. Association of Non-Essential Trace Element Concentrations with Preimplantation IVF Outcomes

The associations between the log-transformed trace element concentrations and preimplantation IVF outcomes were evaluated using models adjusted for age and BMI. The results are presented as the relative proportions’ mean differences (95% CI) between the 20th and 80th percentiles.

Higher FF mercury levels were associated with lower fertilization rates (p20 vs. p80 (95% CI): 0.78 (0.63, 0.96), *p* trend = 0.022), while higher mercury levels in both FF and blood were associated with lower blastocyst rates (p20 vs. p80 (95% CI): 0.68 (0.52, 0.90), *p* trend = 0.008 and 0.63 (0.41, 0.96), *p* trend = 0.034, respectively). Strontium levels in both FF and blood were associated with lower fertilization (p20 vs. p80 (95% CI): 0.76 (0.61, 0.93), *p* trend = 0.011 and 0.76 (0.61, 0.95), *p* trend = 0.016, respectively), blastocyst (p20 vs. p80 (95% CI): 0.68 (0.50, 0.92), *p* trend = 0.015, and 0.71 (0.52, 0.97), *p* trend = 0.031; respectively), and euploidy rates (p20 vs. p80 (95% CI): 0.65 (0.47, 0.88), *p* trend = 0.008, and 0.65 (0.47, 0.92), *p* trend = 0.016, respectively).

**Figure 2:**
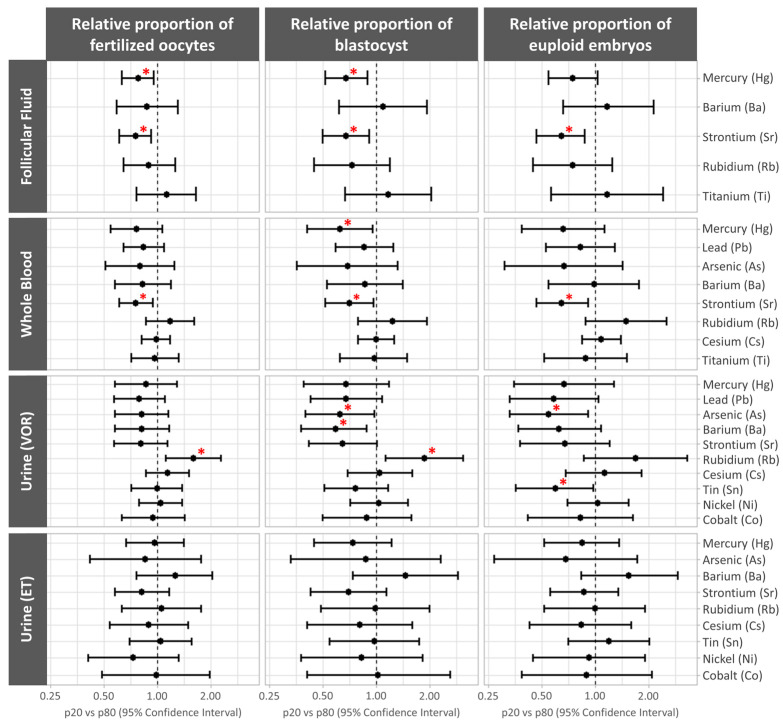
Forest plots representing the mean differences between the 20th and 80th percentiles (p20 vs. p80) (95% confidence interval) for preimplantation IVF outcomes by non-essential trace element concentrations. Mean differences are presented for the relative proportions of oocytes that were successfully fertilized, embryos developing to the blastocyst stage, and euploid blastocysts across the non-essential trace elements quantified in each biological matrix. Data were adjusted for age and BMI (both continuous). * *p* < 0.05. ET, embryo transfer; VOR, vaginal oocyte retrieval.

Elevated levels of urinary arsenic and barium the day of VOR were associated with significantly reduced blastocyst rates (p20 vs. p80 (95% CI): 0.63 (0.40, 0.98), *p* trend = 0.042, and 0.59 (0. 38, 0.89), *p* trend = 0.014; respectively). Meanwhile, elevated levels of urinary arsenic and tin on VOR day were associated with significantly lower euploidy rates (p20 vs. p80 (95% CI): 0.55 (0.33, 0.92), *p* trend = 0.024, and 0.60 (0.36, 0.98), *p* trend = 0.042; respectively). Conversely, elevated levels of urinary rubidium the on VOR day were associated with higher fertilization and blastocyst rates (p20 vs. p80 (95% CI): 1.60 (1.12, 2.29), *p* trend = 0.012, and 1.88 (1.14, 3.09), *p* trend = 0.015; respectively) (Figure 2, Table S2).

### 3.5. Association of Non-Essential Trace Element Concentrations with Clinical IVF Outcomes

Associations between clinical IVF outcomes’ probability with non-essential trace element concentrations in FF, blood, and urine collected both the day of VOR and on the day of ET were evaluated using models adjusted for age and BMI. Data are presented as odds ratios (95% CI).

**Figure 3:**
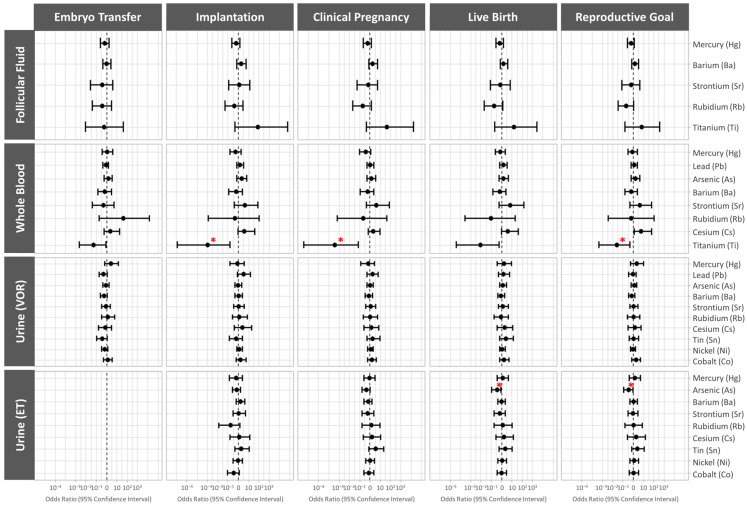
Forest plot representing the odds ratios (95% confidence interval) for clinical reproductive outcomes by non-essential trace element concentrations. The odds ratios are presented for embryo transfer, implantation, clinical pregnancy, live birth, and reproductive goal (achievement of a live birth in a given cycle) following single frozen euploid embryo transfer, across the non-essential trace elements levels quantified in each biological matrix. Data were adjusted for age and BMI (both continuous). In the case of urine obtained on the day of the embryo transfer, the probability of embryo transfer or reproductive success was not examined because it is not informative, since only women who underwent embryo transfer would be considered. * *p* < 0.05. ET, embryo transfer; VOR, vaginal oocyte retrieval.

Participants who underwent embryo transfer and presented elevated levels of whole-blood titanium had significantly lower odds of implantation (OR (95% CI): 0.001 (0.000001, 0.18), *p* value = 0.021) and clinical pregnancy (OR (95% CI): 0.0004 (0.0000004, 0.08), *p*-value = 0.012). Considering all the participants, there were significantly lower odds of achieving a live birth in a given IVF cycle (reproductive goal) when whole-blood titanium was elevated the day of VOR (OR (95% CI): 0.03 (0.0005, 0.5), *p*-value = 0.033) or urinary arsenic levels were elevated the day of embryo transfer (OR (95% CI): 0.37 (0.12, 0.91), *p*-value = 0.047) (Figure 3 and Supplementary Table S3).

## 4. Discussion

Our study’s aim was to investigate whether the concentrations of ten non-essential trace elements in three biological matrices on the day of VOR or embryo transfer were associated with clinical IVF outcomes in Spanish women undergoing ICSI prior to SET/FET with PGT-A. This study was part of a series of investigations where we evaluated the potential implications of non-essential trace elements in IVF treatment in different populations.

In previous studies, we studied associations between non-essential [10] and essential [20] trace element concentrations in similar biological matrices with reproductive outcomes in a cohort of American women who underwent an IVF treatment similar to this population. For the Spanish population, we previously evaluated the impact of essential trace elements on IVF treatment outcomes [19].

In both populations, our study design accounted for confounding factors of IVF treatment, namely maternal age, BMI, ICSI cycles to standardize fertilization procedures, FET cycles to mitigate the potential deleterious effects of COS, and PGT-A to avoid possible implantation failures derived from aneuploidy [29]. Unfortunately, the distinct sociodemographic and lifestyle characteristics of the American and Spanish cohorts and their significantly different reproductive outcomes hindered direct comparison [19].

Below, we focus on the non-essential trace elements that have significant associations with IVF outcomes in Spanish patients.

Corroborating the findings in the American cohort we previously analyzed [10], FF strontium levels were inversely correlated with the relative proportion of mature oocytes retrieved, while FF and whole-blood strontium levels were related to significantly worst pre-implantation outcomes following ICSI. Strontium is an alkaline metal primarily stored in bone because of its similarity to calcium. Notably, steric hindrance prevents strontium from competing with calcium in many biological processes [30]. Humans are mainly exposed to strontium through food and water [30]. There have not been any gonadotoxic effects associated with strontium thus far; its underlying in vivo mechanisms in the reproductive system remain elusive. Strontium chloride is employed to activate oocytes in vitro, enhance fertilization, and induce parthenogenetic activation [31,32]. We hypothesize that environmental overexposure to strontium prematurely activates oocytes, leading to follicular burn-out or incomplete oocyte maturation through similar mechanisms described for other gonadotoxic agents [33]. Additional molecular studies are required to confirm this proposed mechanism.

Mercury is commonly studied in relation to fertility [34] and IVF treatment outcomes. In addition, due to its ability to cross the placental barrier, it can affect different aspects of fetal development, such as neurodevelopment [35]. Metallic, inorganic, and organic forms of mercury contribute to its toxicity [2,34]. Although mercury is naturally deposited in the environment by geological processes, anthropogenic activities are the principal causes of environmental contamination [2,34]. People who consume large quantities of rice, fish, and seafood have a higher risk of mercury poisoning [2,34]. There is growing evidence that mercury overexposure is linked to lower antral follicle counts [16], fewer oocytes retrieved (both total and mature) [16,17], and poor clinical IVF outcomes [8,18]. In this study, we observed that Spanish women with elevated FF mercury concentrations had fewer oocytes retrieved, poor fertilization, and low blastocyst rates. Low blastocyst rates were also observed in Spanish women with elevated whole-blood mercury levels. Notably, other studies did not find such associations [6,7,11,12,13,14,15]. Our previous cohort study [10] showed that mercury levels in FF, plasma, and urine on VOR day were not significantly associated with IVF outcomes. Since FF mercury concentrations were similar between both populations, we hypothesize that, in this Spanish cohort, age may have increased the risk of mercury toxicity [36].

Arsenic is a metalloid present both in organic and inorganic forms. Organic arsenicals (e.g., fish and crustaceans) are considerably less toxic than inorganic arsenicals and their methylated metabolites, which are ingested through contaminated food (e.g., vegetables, fruit, fruit juices, and rice) or water [2]. Corroborating the results of a previous study demonstrating that women overexposed to arsenic had poor embryo quality [37], this study showed that urinary arsenic concentrations the day of VOR were inversely correlated with blastocyst and euploidy rates. Furthermore, we demonstrated that women with high urinary arsenic levels on the day of embryo transfer had lower odds of a live birth, supporting existing evidence that women with low blood arsenic concentrations can achieve pregnancy [38]. In our American cohort study [10], we observed that elevated levels of arsenic in plasma were associated with a suboptimal response to COS, fewer mature oocytes, and low fertilization rates. However, no other associations between arsenic concentrations and IVF treatment outcomes have been reported [7,11,15,17,18]. Given that urine and blood reflect exposure to both forms of arsenic [2], distinguishing inorganic and organic forms in future studies may avoid biases and determine if one form has a more significant impact on reproductive outcomes than the other. A detailed demographic analysis may help reveal lifestyle habits that can be changed to improve the reproductive success of patients undergoing IVF treatment.

Titanium is a ubiquitous element with naturally inert and relatively biocompatible properties, commonly used in dental and medical prosthetics [39,40,41]. Ultrafine titanium dioxide particles are present in cosmetic products, food additives and packaging, and surgical implants, as well as a white pigment [39,40,41]. However, higher levels of titanium have been detected in contaminated food and air pollution [41,42]. Recent evidence suggests that titanium particles are rapidly distributed in the body, and inefficient elimination leads to long-term bioaccumulation, posing risks to human health [39]. Titanium’s ability to cross the placental barrier, among other membranes, raises concerns about its presence during key developmental periods such as pregnancy and lactation [39,42,43]. In our previous study, we found no significant correlations between titanium concentrations in FF, plasma, and urine and IVF outcomes [10]. We have found no other studies evaluating these associations. Here, Spanish women with high whole-blood titanium concentrations were found to have lower odds of implantation, clinical pregnancy, and reproductive success. However, since in the American cohort, the concentration was evaluated in plasma [10], and in this cohort, it has been evaluated in whole blood, the results are not comparable. As titanium appears to be stored mainly intracellularly [44], this may be one of the reasons why no associations were found when assessing titanium levels in plasma. Therefore, we consider that it is better to assess titanium in whole blood rather than in plasma. In murine models, exposure to titanium dioxide during gestation hinders placentation [43,45]. Proposed mechanisms of action include the production of reactive oxygen species [45], autophagy [46], reduced formation of the placental vascular system [47,48], and suppression of embryonic development [49], the latter being consistent with the poor clinical IVF outcomes observed herein. In addition, mice exposed to titanium dioxide before pregnancy presented altered follicle development and poor preimplantation embryo development [50]. Given the evidence of titanium toxicity in animal and human studies, we recommend monitoring titanium levels in IVF cycles to help predict reproductive outcomes.

Barium is an alkaline metal naturally found forming salts, such as sulfates and carbonates [2,51,52]. Although barium salts are used in numerous industrial processes and in some cosmetics, human exposure mainly occurs through contaminated food and water [2,51,52]. In this cohort study, high urinary barium on VOR day was associated with significantly fewer embryos developing to blastocyst stage. Our results are consistent with previous reports of excessive whole-blood barium being associated with reduced oocyte yields, fertilization, and embryo development [15]. In the American cohort [10], high FF barium concentrations were associated with suboptimal responses to COS and poor preimplantation outcomes, while urinary barium concentrations were associated with lower odds of producing a live newborn. Animal models have also shown that overexposure to barium alters ovarian morphology and leads to spontaneous abortions, fetal growth restriction, neonatal defects, and death [51,53]. Despite the lack of negative associations between barium and clinical IVF outcomes in this Spanish cohort, evidence suggests a need to investigate lifestyle-related sources of barium exposure to improve fertility counselling and increase the chances of successful IVF treatment.

Tin can be found as a metal or chemical compound with inorganic and organic elements [54]. Metallic tin is commonly used as a coating for cans (e.g., food, beverages, and aerosols). Inorganic tin compounds are present in cosmetic products and food preservatives, while organic tin compounds (organotins) are employed as pesticides and paint additives and in plastic manufacturing [54,55]. In our previous cohort study [10], we analyzed the association between tin concentrations in various biological fluids and IVF treatment outcomes, discovering that high urinary tin levels were associated with lower odds of achieving a live birth. In Spanish patients, elevated concentrations of tin in VOR day urine were associated with lower euploidy rates. This finding corroborates evidence from animal models showing that organotin exposure impairs oocyte maturation by affecting spindle organization and chromosome alignment, affecting mitochondrial function, inducing oxidative stress, and promoting apoptosis [56,57,58,59]. With the analytical technology used in this study, it was not possible to differentiate between organic and inorganic tin compounds. Future studies should elucidate how different tin forms impact oocyte maturation and competence.

Rubidium is a metal abundantly present in the Earth’s crust [60]. Its dissemination through soil, water, and air favors bioaccumulation in the food chain [60]. Rubidium-rich foods include black tea, coffee, raw fruits and vegetables, poultry meat, and freshwater fish [60]. Given that rubidium excretion occurs mainly through urine, the urinary concentration of rubidium is considered a reliable indicator of exposure [61]. The effects of rubidium on human physiology remain unclear. Until our American cohort study [10], rubidium exposure had not been related to IVF treatment outcomes. We discovered a significant negative association between plasma rubidium and anti-müllerian hormone, an established indicator of the ovarian reserve. While it was not feasible to assess this parameter here, we linked increased urinary excretion of rubidium the day of VOR with significantly higher fertilization and blastocyst rates. A study in goats demonstrated how nutritional rubidium deficiency reduced conception rates and growth, both pre- and postnatal, while drastically increasing the abortion rate (>80%) through insufficient progesterone production [62]. Elevated urinary concentrations of rubidium were also linked to a lower risk of human breast cancer [63], suggesting a potential anti-tumor effect for this element. Together, these findings stress the importance of further investigation on how circulating rubidium affects endocrine regulation and tumor development in reproductive tissues.

Overall, the negative implications of non-essential trace elements discussed here emphasize the importance of broadening the scope of trace element biomonitoring panels to discover new relationships with impaired reproductive function and poor IVF outcomes. Discrepancies between previous studies underscore the need to conduct additional studies in distinct communities to better understand how geographical and sociocultural backgrounds affect exposure. Finally, we acknowledge that, as this was a pilot study, we had a modest sample size, which limits the statistical power of our observations. Additional limitations include that this study did not account for the cumulative results of sequential embryo transfer cycles, and it was not designed to evaluate the impact of paternal exposure, as other groups explored or distinguish between exposure to other environmental pollutants and micronutrients [64,65,66,67,68,69,70].

Therefore, the next steps would be to conduct studies in a larger cohort to simultaneously assess (1) the impact of exposure to non-essential trace elements and other environmental pollutants on IVF outcomes and (2) their modifying effect on micronutrients, like essential trace elements, on IVF-related outcomes.

## 5. Conclusions

Significant negative associations exist between non-essential trace elements and IVF outcomes in a population of Spanish women undergoing a treatment of ICSI with PGT-A and SET/FET. Overall, this study reinforced evidence of the gonadotoxicity of mercury and arsenic, while revealing novel relationships between strontium, titanium, tin, and barium and clinical IVF outcomes. These observations support the need to broaden the scope of non-essential elements investigated in the context of human reproduction. Identifying the mechanisms of action of these elements will help mitigate toxicity, improve fertility counselling, and, ultimately, promote IVF success.

## Figures and Tables

**Table 1:** Baseline characteristics of the participating women (*n* = 51).

**Demographic Characteristics**	
Age (years), median [IQR]	39.00 [38.00, 41.00]
Body mass index (kg/m^2^), median [IQR]	22.97 [20.63, 25.12]
Race/ethnic group, *n* (%)	
- Hispanic-White	48 (94.1%)
- Afro-American	1 (2.0%)
- Hispanic	1 (2.0%)
- Other	1 (2.0%)
Education, *n* (%)	
- Elementary school	3 (6.5%)
- High school	5 (10.9%)
- Trade school	5 (10.9%)
- University	33 (71.7%)
Smoking, *n* (%)	
- Never smoked	23 (50.0%)
- Ex-smoker	13 (28.3%)
- Active smoker	9 (19.6%)
- Passive smoker	1 (2.2%)
**Reproductive Characteristics**	
Total FSH + hMG dose during COS (IU), median [IQR]	3300.00 [2437.50, 3925.00]
Trigger day E2 (pg/mL), median [IQR]	2083.00 [1622.50, 4045.50]
Number of retrieved oocytes, median [IQR]	12.00 [7.00, 16.00]
Maturation rate, % mean ± SD	80 ± 17%
Fertilization rate, % mean ± SD	75 ± 25%
Blastulation rate, % mean ± SD	54 ± 28%
Euploidy rate, % mean ± SD	42 ± 34%
Embryo transfer rate, *n* (%)	36 (70.6%)
Implantation (positive hCG) rate, *n* (%)	23 (63.9%)
Clinical pregnancy rate, *n* (%)	19 (52.8%)
Live birth rate, *n* (%)	17 (47.2%)
Reproductive goal rate, *n* (%)	17 (33.3%)

Note: The reproductive goal rate refers to the achievement of a live birth within a given cycle. COS, controlled ovarian stimulation; E2, estradiol; FSH, follicle-stimulating hormone; hCG, human chorionic gonadotropin; hMG, human menopausal gonadotropin; IQR, interquartile range; n, number; SD, standard deviation.

**Table 2:** Distribution of non-essential trace element concentrations in follicular fluid, whole blood, and urine obtained the day of vaginal oocyte retrieval and urine obtained the day of embryo transfer.

	LOD	Samples < LOD, *n* (%)	GM (SD)	20%	50%	80%
**Follicular Fluid (*n* = 29)**						
Mercury (Hg) (ng/mL)	0.5	4 (10%)	1.96 (2.59)	1.31	2.12	3.73
Lead (Pb) (ng/mL)	1	29 (74%)	0.63 (0.50)	0.50	0.50	0.50
Arsenic (As) (ng/mL)	1	19 (49%)	1.02 (1.30)	0.50	1.00	2.04
Barium (Ba) (ng/mL)	1	2 (5%)	2.73 (5.15)	1.16	2.60	6.02
Strontium (Sr) (ng/mL)	NA	0 (0%)	33.68 (11.84)	26.81	35.00	43.00
Rubidium (Rb) (ng/mL)	NA	0 (0%)	83.30 (31.69)	62.80	93.62	113.23
Titanium (Ti) (ng/mL)	NA	0 (0%)	2.48 (0.50)	2.14	2.39	3.00
**Whole Blood (*n* = 40)**						
Mercury (Hg) (ng/mL)	NA	0 (0%)	6.21 (5.19)	3.78	6.01	10.89
Lead (Pb) (ng/mL)	1.5	6 (13%)	4.93 (20.87)	2.52	5.58	9.31
Arsenic (As) (ng/mL)	1.5	19 (40%)	1.76 (1.77)	0.75	1.97	3.66
Barium (Ba) (ng/mL)	1.5	2 (4%)	2.58 (1.43)	1.83	2.37	4.14
Strontium (Sr) (ng/mL)	NA	0 (0%)	28.13 (9.31)	23.51	28.77	34.44
Rubidium (Rb) (ng/mL)	NA	0 (0%)	1205.68 (151.40)	1073.35	1233.01	1304.84
Cesium (Cs) (ng/mL)	1.5	2 (4%)	2.89 (6.93)	2.07	2.63	3.55
Titanium (Ti) (ng/mL)	NA	0 (0%)	5.26 (1.65)	4.23	5.18	6.17
Nickel (Ni) (ng/mL)	1.5	32 (67%)	1.47 (7.15)	0.75	0.75	4.12
**Urine, VOR (*n* = 50)**						
Mercury (Hg) (ng/mL)	NA	0 (0%)	1.90 (0.94)	1.29	2.01	2.66
Creatinine-corrected Hg (µg/g CR)			0.48 (0.25)	0.32	0.49	0.75
Lead (Pb) (ng/mL)	0.5	15 (30%)	0.64 (2.20)	0.25	0.64	0.99
Creatinine-corrected Pb (µg/g CR)			0.16 (0.67)	0.08	0.15	0.27
Arsenic (As) (ng/mL)	NA	0 (0%)	51.8 (109.67)	24.10	53.72	113.49
Creatinine-corrected As (µg/g CR)			12.78 (27.35)	4.82	13.54	28.39
Barium (Ba) (ng/mL)	0.5	5 (10%)	1.93 (3.47)	0.75	2.37	4.53
Creatinine-corrected Ba (µg/g CR)			0.48 (1.22)	0.19	0.52	0.99
Strontium (Sr) (ng/mL)	NA	0 (0%)	227.32 (217.26)	105.32	269.76	497.51
Creatinine-corrected Sr (µg/g CR)			55.27 (47.92)	29.68	57.29	92.79
Rubidium (Rb) (ng/mL)	NA	0 (0%)	842.46 (508.08)	545.43	830.17	1357.96
Creatinine-corrected Rb (µg/g CR)			211.86 (108.70)	137.13	206.19	315.74
Cesium (Cs) (ng/mL)	NA	0 (0%)	5.98 (3.16)	4.06	6.12	8.18
Creatinine-corrected Cs (µg/g CR)			1.49 (0.90)	1.07	1.46	1.95
Tin (Sn) (ng/mL)	0.5	1 (2%)	1.80 (1.78)	1.26	1.84	2.39
Creatinine-corrected Sn (µg/g CR)			0.44 (0.37)	0.28	0.48	0.71
Nickel (Ni) (ng/mL)	1	4 (8%)	3.07 (11.62)	1.60	2.50	7.09
Creatinine-corrected Ni (µg/g CR)			0.75 (4.04)	0.34	0.59	1.60
Cobalt (Co) (ng/mL)	0.5	23 (46%)	0.52 (0.71)	0.25	0.51	1.14
Creatinine-corrected Co (µg/g CR)			0.13 (0.16)	0.06	0.14	0.23
**Urine, ET (*n* = 27)**						
Mercury (Hg) (ng/mL)	NA	0 (0%)	1.67 (2.93)	1.00	1.73	2.26
Creatinine-corrected Hg (µg/g CR)			0.51 (0.81)	0.32	0.50	0.75
Lead (Pb) (ng/mL)	0.5	16 (57%)	0.45 (1.31)	0.25	0.25	0.81
Creatinine-corrected Pb (µg/g CR)			0.14 (0.53)	0.07	0.12	0.25
Arsenic (As) (ng/mL)	NA	0 (0%)	31.89 (68.56)	8.73	40.45	73.65
Creatinine-corrected As (µg/g CR)			9.84 (17.37)	3.28	9.12	28.88
Barium (Ba) (ng/mL)	0.5	3 (11%)	1.41 (2.92)	0.69	1.31	3.43
Creatinine-corrected Ba (µg/g CR)			0.44 (0.75)	0.18	0.44	0.98
Strontium (Sr) (ng/mL)	NA	0 (0%)	139.16 (120.93)	90.61	164.24	224.32
Creatinine-corrected Sr (µg/g CR)			42.96 (33.66)	28.94	46.21	70.08
Rubidium (Rb) (ng/mL)	NA	0 (0%)	792.90 (487.88)	494.65	807.54	1115.83
Creatinine-corrected Rb (µg/g CR)			244.75 (107.57)	172.66	261.40	351.82
Cesium (Cs) (ng/mL)	NA	0 (0%)	5.42 (2.16)	3.92	5.76	7.43
Creatinine-corrected Cs (µg/g CR)			1.67 (0.91)	1.21	1.58	2.17
Tin (Sn) (ng/mL)	NA	0 (0%)	1.45 (2.37)	0.90	1.31	1.75
Creatinine-corrected Sn (µg/g CR)			0.45 (0.56)	0.26	0.41	0.63
Nickel (Ni) (ng/mL)	0.5	1 (4%)	1.93 (2.50)	1.08	2.50	2.50
Creatinine-corrected Ni (µg/g CR)			0.60 (0.90)	0.27	0.56	1.33
Cobalt (Co) (ng/mL)	0.5	12 (43%)	0.53 (0.63)	0.25	0.55	1.14
Creatinine-corrected Co (µg/g CR)			0.16 (0.17)	0.07	0.15	0.38

CR, creatinine; GM, geometric mean; LOD, limit of detection; NA, not applicable; SD, standard deviation; ET, embryo transfer; VOR, vaginal oocyte retrieval.

## Data Availability

The data presented in this study are openly available in Mendelei.

## Supplementary Materials

The following supporting information can be downloaded at https://www.mdpi.com/article/10.3390/jox14030062/s1. Figure S1: Spearman correlation matrix representing the relationships of non-essential trace elements between biological matrices. Figure S2: Spearman correlation matrix representing the relationships between the non-essential trace elements within each biological matrix. Table S1: Mean differences (95% confidence interval) in ovarian response-related outcomes by non-essential trace element concentrations. Table S2: Mean differences (95% confidence interval) in the preimplantation IVF outcomes by non-essential trace element concentrations. Table S3: Odds Ratios (95% Confidence Interval) for the clinical reproductive variables by non-essential trace element concentrations.
