# Supplementary figures and images for: Associations between Non-Essential Trace Elements in Women’s Biofluids and IVF Outcomes in Euploid Single-Embryo Transfer Cycles
Source: J Xenobiot. 2024 Aug 8;14(3):1093–108. doi: 10.3390/jox14030062 (PMC11348048; doi:10.3390/jox14030062)

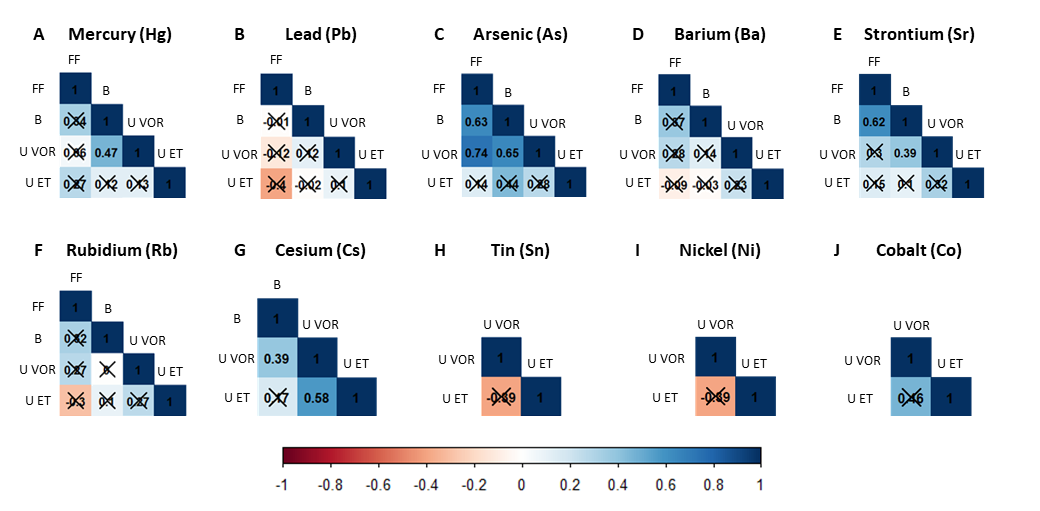

Supplement: Supplementary file 1 [file jox-14-00062-s001.zip › Fig S1 - Correlations metabolite NonEssential.TIF]

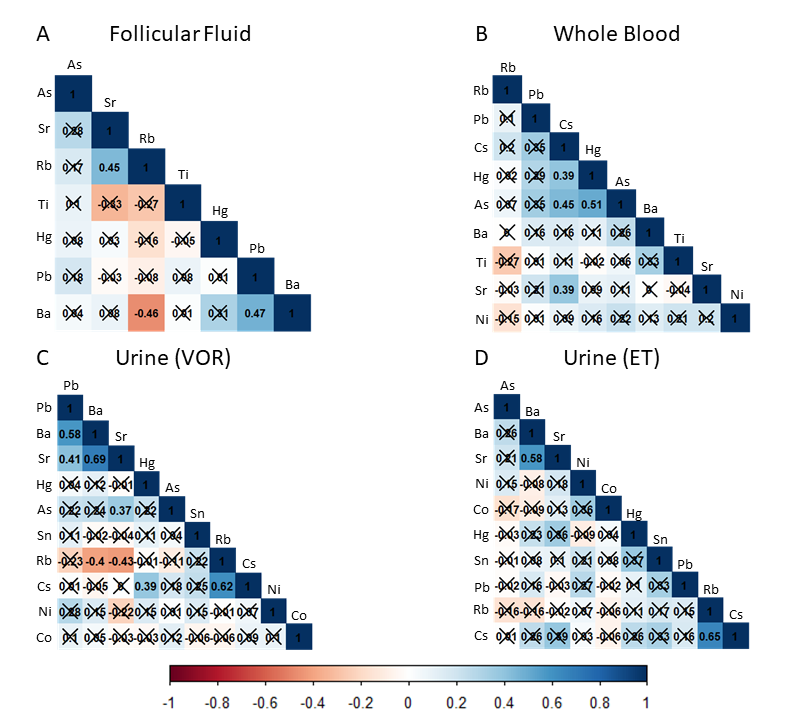

Supplement: Supplementary file 1 [file jox-14-00062-s001.zip › Fig S2 - Correlations matrix NonEssential.TIF]
